# The Scottish medicines in pregnancy programme: supporting the surveillance of medicines use during pregnancy and assessment of associated clinical outcomes

**DOI:** 10.1007/s00228-026-04062-2

**Published:** 2026-04-24

**Authors:** Tanja Mueller, Laura Stobo, Lynne Jarvis, Morven Millar, Emily Moore, Leanne Hopkins, Victoria Stark, Amy Hynd, Kwaku Osei-Oppong, Amanj Kurdi, Stuart McTaggart, Rachael Wood, Marion Bennie

**Affiliations:** 1 Strathclyde Institute of Pharmacy and Biomedical Sciences, University of Strathclyde, Glasgow, UK; 2 Public Health Scotland, Edinburgh, UK; 3 NHS Greater Glasgow & Clyde, Glasgow, UK; 4 Usher Institute, University of Edinburgh, Edinburgh, UK

**Keywords:** Medicines, Pregnancy, Safety, Surveillance, Scotland

## Abstract

**Purpose:**

Pharmacological treatment during pregnancy is often necessary but presents challenges due to limited availability of safety data. In response to a UK-wide review of medicine safety, the Scottish Government commissioned the development of a national surveillance infrastructure to monitor medicines use during pregnancy and associated clinical outcomes.

**Methods:**

Two real-world data assets were developed by integrating routinely collected primary and secondary care data: the Scottish Combined Medicines Dataset (SCoMeD) consolidates medicines data, while the Scottish Linked Pregnancy and Baby Dataset (SLiPBD) captures comprehensive data related to pregnancies. Two exemplar case studies were conducted to test the utility of these datasets. First, an audit of anti-seizure medicines (ASM) prescribing 04.2018–03.2025; and second, a retrospective cohort study of pregnancies conceived between 04.2010 and 06.2023, matching ASM exposed to ASM unexposed pregnancies to assess pregnancy, baby, and early childhood outcomes. Study periods were chosen based on data availability.

**Results:**

The linkage of SCoMeD and SLiPBD enabled national-level surveillance of ASM prescribing trends and in-utero exposures. ASM prescribing surveillance showed an expected decrease in the use of valproate and topiramate among women of childbearing age, reflecting regulatory risk minimisation measures. Observational analyses confirmed valproate-associated teratogenic and developmental risks, whilst supporting the relative safety of lamotrigine and levetiracetam during pregnancy.

**Conclusions:**

This programme demonstrates the value of leveraging nation-wide, whole system data for the surveillance of medicines safety in pregnancy. The linkage of real-world data provides regulatory-relevant evidence to inform guidelines and risk minimisation strategies. Scotland’s integrated medicines infrastructure positions the country as a key contributor in the European pharmacovigilance landscape.

## Key points

• There is considerable uncertainty regarding the safety of medicines during pregnancy due to pregnant women being frequently excluded from clinical trials.

• Two new substantive data resources have been developed in Scotland to enable the national surveillance of medicine use during pregnancy and support ongoing efforts to de-risk regulatory decision-making.

• The Scottish Combined Medicines Dataset (SCoMeD) and the Scottish Linked Pregnancy and Baby Dataset (SLiPBD) provide consolidated patient-level medicines data from both primary and secondary care and detailed pregnancy information, respectively, and can be linked to other national datasets to facilitate population-based pregnancy pharmacoepidemiology research.

## Introduction

Medicines are the most widely used intervention in healthcare and are usually considered a safe and effective means of treating or managing health conditions; however, in certain situations, pharmacological treatment may become a complex topic – for instance, during pregnancy.

While the mother’s health is important for the baby’s health and pregnant women may need to take medication to ensure disease control – as an example, to control seizures in women with epilepsy – there are two main challenges related to medicines use during pregnancy. First, several medicines can adversely affect pregnancy outcomes or cause structural congenital or developmental conditions. For instance, the teratogenic potential of valproate is widely known [1, 2], and safety concerns have been raised for other anti-seizure medications (such as topiramate) as well as a range of additional medicines used to treat a variety of conditions, including retinoids used in dermatology [3, 4]. Second, there is still considerable uncertainty regarding the safety of medicines during pregnancy more generally – not least due to pregnant women being commonly excluded from clinical trials [5]. Furthermore, observational studies aimed at providing real-world evidence on the effectiveness and safety of medicines use during pregnancy face significant challenges, spanning from low sample sizes to difficulties related to confounding [6, 7]. Variations in study designs, populations, data collection, and analytical methods also mean that findings from different studies may be neither directly comparable nor generalisable [8, 9].

To at least partially address these existing challenges, collecting data relating to the safety of medicines during pregnancy is standard practice in many countries. Pregnancy and disease registries, alongside the routine collection of data through reporting schemes for suspected adverse effects – such as the United States Food and Drug Administration (US FDA) Adverse Event Reporting System (FAERS) [10], the EudraVigilance system of the European Union [11], or the Yellow Card Scheme in the UK [12] – have long been established. In addition, organisations such as the US FDA and the European Medicines Agency (EMA) have developed guidelines on post-authorisation data collection during pregnancy [13, 14].

Nevertheless, the UK-wide Independent Medicines and Medical Devices Safety (IMMDS) review, commissioned in 2018, has identified a number of areas for improvement [15]. Central among the recommendations made in the subsequently published report, “First do no harm”, is the call for comprehensive, robust, and reusable data on healthcare use, interventions, and outcomes [16]. This specifically included setting up a database of all women of child-bearing potential being prescribed sodium valproate.

To implement the recommendations of the IMMDS review, the Scottish Government developed a Delivery Plan [17] that informed the establishment of the Teratogenic Medicines Advisory Group (TMAG) [18]. In 2022, TMAG commissioned Public Health Scotland (PHS) to deliver a national medicines surveillance system in relation to pregnancy, with the aims of producing a whole system medicines data asset (i.e., a dataset capturing patient-level data on utilisation of medicines across the whole healthcare system, including both primary and secondary care), initially focusing on anti-seizure medicines (ASMs); creating a dynamic pregnancy and related outcomes dataset; and facilitating linkage between the medicines and pregnancy datasets, with the possibility of linking further datasets.

## Methods

Supported by funding from the Scottish Government, a project team was formed within PHS comprised of pharmacists, public health specialists, epidemiologists, and data scientists; additional expertise was provided by a TMAG subgroup that included neurologists, obstetricians, specialist pharmacists, and representatives from both the voluntary sector as well as the Scottish Government’s medicines policy team. This newly formed Medicines in Pregnancy team set out to achieve the aforementioned objectives between 2022 and 2024.

### Overview of the Scottish health care system

Scotland – a country with a population of approximately 5.5 million people [19], similar in size to Finland (5.6 million), Norway (5.6 million), or Denmark (6 million) [20] – has universal health care. The Scottish National Health Service (NHS) is tax-funded, and services, including prescription medicines, are free of charge at the point of care. More specifically, NHS Scotland provides pregnancy, labour, and postnatal care; this includes regular appointments with a midwife or an obstetrician throughout pregnancy, and also involves the prescribing of medicines as required, in line with existing guidelines [21]. Every resident is allocated a unique patient identifier, the Community Health Index (CHI) number, either at birth or at the time of first registration with the NHS, which is used throughout the healthcare system and is included – with some minor exceptions – in all health records [22]. Over the last decade, efforts have been made to digitise the system in line with the Scottish Government’s Digital Strategy [23], and electronic prescribing has been implemented in both primary and secondary care settings across Scotland.

### Approach to achieving the objectives as set out by the Teratogenic Medicines Advisory Group

In a first step, the availability of patient-level data across Scotland was assessed; plans were then developed to bring together existing datasets in order to establish both a national whole system medicines asset and a dynamic pregnancy and related outcomes dataset. Variable selection was informed primarily by clinical and analytical requirements and guided by information governance principles [24]; usability was assured through continuous advice from technical experts and several rounds of user testing, which comprised pilot data extraction, checking of data completeness, and assessing extracted data based on expectations to ensure that data was in line with technical specifications as well as content requirements. Following the implementation of these two new resources, exemplar case studies were conducted to test the linkage between the medicines and pregnancy datasets, with some initial piloting of linkage to additional datasets (e.g., hospital inpatient episodes). Record linkage was facilitated using CHI numbers.

### Landscape of data availability in Scotland

Patient level prescribing data for medicines supplied in primary care in Scotland has been available since April 2009 from the Prescribing Information System (PIS) [25]. Secondary care patient level medicines data has also become available in recent years through two new national prescribing datasets: the Hospital Electronic Prescribing and Medicines Administration (HEPMA) system, covering hospital inpatient prescribing from July 2022 onwards [26]; and the Homecare Medicines (HCM) dataset, covering specialist medicines prescribed in hospital and delivered directly to a patient’s home since January 2019 [27]. To produce a whole system medicines intelligence asset, the aim was to combine these three datasets (PIS, HEPMA, HCM) and create the Scottish Combined Medicines Dataset (SCoMeD). Methodological details regarding the development of SCoMeD can be found elsewhere [28].

Similar to pre-existing data on medicines, several datasets relating to pregnancy and births have been available in Scotland. For example, episode-level data for obstetric events have been collected in the Scottish Morbidity Records Maternity Inpatient and Day-case (SMR02) dataset since 1975 [29]; similarly, National Records of Scotland (NRS) Births, Stillbirths, and Infant Deaths records have been available through PHS and its predecessors since 1975 [30]. The national antenatal bookings dataset is available from 2019 onwards. To increase efficiency and accuracy of analyses, the aim was to combine existing datasets and create the Scottish Linked Pregnancy and Baby Dataset (SLiPBD). Methodological details regarding the development of SLiPBD can be found elsewhere [31].

### Exemplar case studies

Following the development of SCoMeD and SLiPBD, two initial projects were conducted to pilot record linkage and test data capabilities: first, an audit of ASM prescribing, with a special focus on prescribing during pregnancy; and second, a retrospective observational study on pregnancy, baby, and early childhood outcomes of ASM treatment during pregnancy.

To describe prescribing of ASMs – defined as any medicine included in Chap. 4.8 “antiseizure medicines” of the British National Formulary (BNF), the most commonly used medicines classification system in the UK [32] – data were extracted from SCoMeD, and prescribing trends since 2018 were summarised by age group, initially for all female patients up to the age of 54 years and subsequently also for male patients covering the same age groups. Medicines records were then linked to SLiPBD to provide an overview of prescribing during pregnancy and summarise in utero exposure to ASMs [33]. The start date of the audit was informed by the publication of advice to not prescribe valproate during pregnancy, issued by the Medicines and Healthcare products Regulatory Agency (MHRA) in April 2018 [34], whereas the end date was determined by data availability at the time of writing (June 2025). The subsequent study on treatment outcomes used a retrospective cohort study design, matching ASM exposed to ASM unexposed pregnancies. Exposure was again defined as medicines included in BNF Chap. 4.8; outcomes of interest included pregnancy loss, major structural congenital conditions, and early childhood developmental concerns. The latter were defined as any concern recorded against any developmental domain (speech, language and communication; gross motor; fine motor; problem solving: personal/social; emotional/behavioural) at the 27–30 month assessment routinely offered to all children in Scotland [35]. In addition to SCoMeD (ASM prescribing data) and SLiPBD (information on pregnancies and pregnancy outcomes), datasets including the Scottish Linked Congenital Conditions Dataset (SLiCCD) and the Child Health Systems Programme: Pre-School (CHSP-PS) dataset were linked via CHI numbers. Similar to the audit, the cohort study period was chosen based on data availability at the time of study protocol development, taking into account the earliest time point from which prescribing data can be reliably linked to pregnancy data (2010) as well as the minimum follow-up times required to assess treatment outcomes. For details, see the study protocol [36].

## Results

Sparked by the IMMDS report and set in motion by the call to set up a surveillance system in relation to pregnancy, the Medicines in Pregnancy team at PHS has delivered on all three objectives: produce a national, whole system medicines asset; create a dynamic pregnancy and related outcomes dataset; and facilitate linkage between the medicines and pregnancy datasets. The feasibility of linkage with additional dataset has also been established.

### Developed data assets

By combining individual-level medicines records from both primary and secondary care settings, the SCoMeD resource gives a unified view of all medicines data, providing an up-to-date longitudinal dataset of medicines use for patients treated within NHS Scotland, regardless of where these medicines have been prescribed. SCoMeD captures information on medicine name, strength, quantities, and dates of prescribing/dispensing (primary care) or administration (secondary care) [28].

The creation of SLiPBD provides a population-based e-cohort of all foetuses and babies (births) stemming from pregnancies in Scotland from 2000 onwards, updated on a monthly basis and providing complete information on pregnancies conceived or completed up to three months previously. SLiPBD includes information on estimated date of conception, gestation, and pregnancy outcome [31].

Both SCoMeD and SLiPBD have since been adopted for use within NHS Scotland. While SLiPBD is available upon request to researchers through the Electronic Data Research and Innovation Service (eDRIS) [37], work is ongoing to make SCoMeD available to researchers.

### Routine clinical surveillance of ASM prescribing

Surveillance data on ASM prescribing in Scotland are now routinely published through a publicly available dashboard, accompanied by summary reports on the PHS website [38]; a more in-depth management resource (i.e., a detailed spreadsheet), providing granular geographical breakdowns of ASM prescribing and exposure to ASMs during pregnancy, is made available to NHS Scotland staff.

The inaugural *Anti-seizure medicines use in Pregnancy* report included data from April 2018 to September 2023 [39], and dashboard updates are now released every six months. From April 2018 to March 2025, the total number of female patients aged 0–54 years captured in the data as having been prescribed an ASM is 166,723; overall, 7,413 pregnancies (to 5,927 women) were exposed to an ASM between April 2018 and March 2025 [38].

**Fig. 1:**
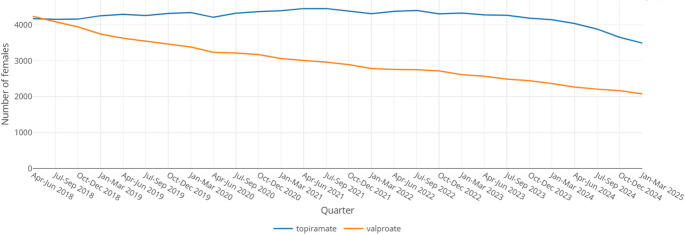
Number of female patients aged 0–54 years prescribed valproate and topiramate in Scotland, April 2018 – March 2025 [38]

Due to specific warnings issued by the MHRA, the focus of the *ASM in pregnancy* dashboard are valproate and topiramate (for any indication). For both medicines, pregnancy prevention programmes (PPPs) are in place in the UK; the valproate PPP was introduced in April 2018, and the topiramate PPP in June 2024 [40, 41]. Prescribing for valproate has decreased by 51% in female patients aged 0–54 years since April 2018; prescribing for topiramate has also started to decrease with the introduction of the respective PPP (Fig. 1).

Most recently, warnings regarding reproductive risks of valproate have been extended to its use in male patients based on observational studies indicating a possibly increased risk of neurodevelopmental disorders in children born to fathers being treated with valproate in the three months prior to conceptions [42]. Accordingly, updated advice has been introduced in the UK in January 2024 [43]. Due to these developments – and facilitated by the dynamic nature of the medicines dataset – prescribing of ASMs in male patients was added to the dashboard, for the first time, in April 2025. Since the introduction of the new advice, prescribing of valproate in male patients aged 0–54 years has been reduced by 12% [38]. For full details on the prescribing of ASMs and the exposure to ASMs during pregnancy, see the publicly available dashboard [38].

### Retrospective cohort study assessing pregnancy, baby, and early childhood outcomes

The population-based cohort study comprised 911,027 singleton pregnancies of which 11,011 were exposed to ASMs. Amongst the exposed pregnancies, 28.8% experienced pregnancy loss (either spontaneous loss or termination); 2.7% of births/foetuses were affected by a congenital condition. Early childhood developmental concerns were raised in 26.0% of children with sufficient follow-up time and complete records. While both valproate and topiramate showed a statistically significant association with pregnancy loss after adjusting for confounders, only valproate was significantly associated with congenital conditions. Valproate was also associated with developmental concerns. The study publication provides further details [44].

### Further work

Additional analyses are in progress to further test data capabilities, including an observational study focusing on ASM polytherapy during pregnancy and an evaluation of the safety of high-dose folic acid. Furthermore, work is ongoing to identify additional areas where the developed surveillance system may be deployed in the future – for instance, the safety of biologics in pregnancy.

## Discussion

The Medicines in Pregnancy programme has successfully developed and adopted a system that facilitates the national surveillance of medicines use in pregnancy utilising data routinely collected in clinical care, in line with plans set out in the Scottish Government’s Delivery plan in the wake of the IMMDS report [17]. With two major new data resources in place – SCoMeD and SLiPBD, providing comprehensive data related to medicines and pregnancy, respectively – medicines surveillance outputs such as public medicines dashboards and management reports can now routinely be produced and updated in line with emerging requirements, as evidenced by the rapid inclusion of valproate prescribing to male patients following a change in clinical guidelines. Furthermore, the developed system has in-build capacity to expand to other medicines of interest, thereby offering insights into the use of potentially teratogenic medicines besides ASMs. This is directly in line with calls by regulatory agencies across the UK to ensure the development of appropriate tools and interventions to minimise harm to patients [15, 17].

While requirements regarding the collection of data pertaining to patient safety (especially during pregnancy) can be found in other countries, specific approaches to data collection and use differ considerably. A common method to ensure that pregnancy safety data is readily available for analysis is the setting up of dedicated pregnancy registries, such as the North American Anti-epileptic drug pregnancy register or the Australian Pregnancy Register [45, 46]. Although these pregnancy registers provide comprehensive, high-quality data, they were developed to cover specific patient groups or treatments of interests, and data on other medicines that were either not of concern or were not available at the time of registry implementation may not be included. Another approach to obtaining pregnancy safety data is the collection of spontaneous reports of adverse events; various schemes exist globally [10–12]. These schemes rely on active participation of healthcare professionals including physicians and pharmacists and require awareness of both medication side effects and reporting schemes by patients, with under-reporting of adverse events considered an important limitation [47]. Prospective studies such as post-authorisation safety studies (PASS), which may or may not be mandated following market approval of a new medicine, can also be used to obtain information on a medicine’s safety; however, PASS can be costly and time-consuming, and may not always be practical – especially if a medicine has already been on the market for some time [48, 49].

In contrast, using data routinely collected in clinical care to assess medicine safety offers the advantage of providing information on any medicine of interest, provided that medicines data is comprehensive, accurate, and timely – and, ideally, attributable to individual patients so as to support analyses of not only prescribing but also ensuing treatment outcomes. The continuously evolving array of advanced epidemiological methods available to researchers, for instance target trial emulation [50], further facilities causal inference on medicine effectiveness and safety through observational studies in circumstances where RCTs are unfeasible.

The Nordic countries Denmark, Norway, and Sweden have long been considered the gold-standard with regards to population-based registries [51], setting the benchmark for pregnancy pharmacoepidemiology as a source for our current knowledge of the harmful effects of ASMs [52]. Following the tradition of the Nordic countries, the main strengths of the surveillance capacities that have now been put in place in Scotland are the comprehensiveness and high quality of the available data. Nevertheless, the Scottish infrastructure also offers several distinct advantages. A key distinguishing feature is the integration of routinely collected patient-level data from primary care, secondary care, and specialist homecare medicines services in a single dataset. SCoMeD offers a near-complete picture of medicines across the entire healthcare system, including high-cost and hospital-initiated therapies often missing from prescribing datasets internationally. In parallel, SLiPBD offers high-quality information on all recognised pregnancies, with robust capture of all outcomes including live births, stillbirths, miscarriages, and terminations of pregnancy – the latter two often being underreported or missing from datasets. Although the lack of indication for prescribing within the medicines dataset is a current limitation of the resources, the presence of a unique patient identifier in Scotland, in tandem with the wide range of potentially linkable datasets, provides the distinctive opportunity to evaluate prescribing patterns and trends during pregnancy as well as to analyse treatment outcomes.

## Conclusion

The establishment of national surveillance assets in Scotland demonstrates how routinely collected, linked health data can be harnessed to deliver robust, real-world evidence on medicines use and safety during pregnancy, thus contributing meaningfully to the European pharmacovigilance and pregnancy safety surveillance landscape. With a population comparable to Nordic countries, long recognised for their registry-based pharmacoepidemiology, Scotland now possesses a robust infrastructure for linking longitudinal medicines and pregnancy outcome data at the population level. These capabilities support proactive risk–benefit assessments of medicines used during pregnancy and facilitate real-world evidence generation to inform regulatory decision-making, aligned with the priorities of agencies such as FDA and EMA to de-risk regulatory decision making through enhanced use of real-world data.

## Data Availability

The data underlying this article cannot be shared publicly due to ethical reasons; access to pseudonymised individual-level healthcare data in Scotland is subject to approval by the Public Benefit and Privacy Panel for Health and Social Care.
